# Clinical verification of ^18^F-DCFPyL PET-detected lesions in patients with biochemically recurrent prostate cancer

**DOI:** 10.1371/journal.pone.0239414

**Published:** 2020-10-06

**Authors:** Dennie Meijer, Bernard H. E. Jansen, Maurits Wondergem, Yves J. L. Bodar, Sandra Srbljin, Annelies E. Vellekoop, Bram Keizer, Friso M. van der Zant, Otto S. Hoekstra, Jakko A. Nieuwenhuijzen, Max Dahele, André N. Vis, Daniela E. Oprea-Lager

**Affiliations:** 1 Department of Urology, Prostate Cancer Network Amsterdam, Amsterdam University Medical Centers, VU University, Amsterdam, The Netherlands; 2 Department of Radiology & Nuclear Medicine, Cancer Center Amsterdam, Amsterdam University Medical Centers, VU University, Amsterdam, The Netherlands; 3 Department of Nuclear Medicine, Noordwest Ziekenhuisgroep, Alkmaar, The Netherlands; 4 Department of Nuclear Medicine, Zaans Medical Center, Zaandam, The Netherlands; 5 Department of Urology, Amstelland Hospital, Amstelveen, The Netherlands; 6 Department of Urology, Dijklander Hospital, Hoorn, The Netherlands; 7 Department of Radiation Oncology, Cancer Center Amsterdam, Amsterdam, The Netherlands; National Health Research Institutes, TAIWAN

## Abstract

**Purpose:**

Radiolabeled Prostate-Specific Membrane Antigen (PSMA) PET/CT is the current standard-of-care for lesion detection in patients with biochemically recurrent (BCR) prostate cancer (PCa). However, rigorous verification of detected lesions is not always performed in routine clinical practice. To aid future ^18^F-radiolabeled PSMA PET/CT interpretation, we aimed to identify clinical/imaging characteristics that increase the likelihood that a PSMA-avid lesion is malignant.

**Materials and methods:**

262 patients with BCR, who underwent ^18^F-DCFPyL PSMA PET/CT, were retrospectively analyzed. The malignant nature of ^18^F-DCFPyL PET-detected lesions was verified through any of the following metrics: (1) positive histopathological examination; (2) additional positive imaging; (3) a ≥50% decrease in Prostate-Specific Antigen (PSA) following irradiation of the lesion(s).

**Results:**

In 226/262 PET scans (86.3%) at least one lesion suspicious for recurrent PCa was detected (‘positive scan’). In 84/226 positive scans (37.2%), at least one independent verification metric was available. PSMA PET-detected lesions were most often confirmed to be malignant (PCa) in the presence of a CT-substrate (96.5% vs. 55.6% without CT-substrate), with SUV_peak_ ≥3.5 (91.4% vs. 60.0% with SUV_peak_<3.5), in patients with a PSA-level ≥2.0 ng/mL (83.7% vs. 65.7% in patients with PSA <2.0ng/mL) and in patients with >2 PET-positive lesions (94.1% vs. 64.2% in patients with 1–2 PET-positive lesions; *p*<0.001–0.03).

**Conclusions:**

In this study, the clinical verification of ^18^F-DCFPyL PET-positive lesions in patients with BCR was performed. Diagnostic certainty of PET-detected lesions increases in the presence of characteristic abnormalities on CT, when SUV_peak_ is ≥3.5, when PSA-levels exceed 2.0 ng/mL or in patients with more than two PET-positive lesions.

## Introduction

In the Western world, prostate cancer (PCa) is the most common cancer in men [1, 2]. Localized PCa is mostly treated by means of radical prostatectomy (RP), external beam radiotherapy (EBRT) or brachytherapy (BT) [3]. Although these treatment options are curative in intent, approximately 20–40% of all patients develop a biochemical recurrence (BCR) of PCa after RP or EBRT [4–8].

Positron Emission Tomography/Computed Tomography (PET/CT) using Prostate-Specific Membrane Antigen (PSMA) targeting radiotracers, is increasingly used for PCa diagnostics. PSMA is a transmembrane protein that provides an important target for radiolabeled imaging as its expression is upregulated in malignant prostate cells. Furthermore, it is correlated with higher tumor grades, as well as with risk of disease progression [9–11]. PSMA PET has shown promising results for detecting metastases in patients with BCR and is recommended in current clinical guidelines [12–14].

For the localization of BCR, ^68^Gallium (^68^Ga)-labeled PSMA tracers have been studied intensively [14–17]. ^18^Fluorine (^18^F)-labeled PSMA tracers have also been developed, for example ^18^F-DCFPyL, a second generation ^18^F-labeled PSMA-targeted radiotracer [18]. ^18^F-DCFPyL has shown higher tumor to background ratios compared to ^68^Ga-PSMA [19]. Furthermore, the ^18^F-radionuclide provides better PET-image resolution than ^68^Ga, due to a shorter positron range and higher positron yield, which may improve early detection of small metastases [20, 21].

In patients with BCR, the detection rate of PSMA PET/CT is promising (^68^Ga-PSMA 80–85%; ^18^F-DCFPyL 81–86%) [13, 22–24]. However, rigorous verification of PSMA PET-detected lesions is seldom performed, often because histological verification (biopsy) of small lesions is not feasible or considered clinically appropriate. In practice, the malignant nature of PSMA PET-detected lesions that can also be seen on anatomical imaging (e.g. CT, MRI) is often presumed. However, because the presence of PSMA-avid lesions may lead to the initiation of treatment, e.g. (oligo)metastases-directed (stereotactic) radiotherapy, salvage lymph-node dissection, or systemic treatment, false positive lesion detection could lead to overtreatment. Therefore, the aim of this study was to evaluate ^18^F-DCFPyL PET-detected lesions in patients with BCR and identify clinical/imaging characteristics that increase diagnostic certainty regarding the malignant nature of the lesions.

## Materials and methods

262 consecutive patients with BCR, scanned with ^18^F-DCFPyL PET/CT between November 2016 and March 2019 in two Dutch PCa reference centers (Amsterdam University Medical Center, VU University Medical Center (n = 83); Noordwest Ziekenhuisgroep Alkmaar (n = 179)), were retrospectively analyzed. Part of this cohort was already included in an analysis strictly focusing on lesion localization [13], whereas the current study has its main focus on lesion verification. The study was approved by the institutional review board of the VU University Medical Center, which waived the need to obtain informed consent (review number 2019.275). All data obtained from the patient records was pseudonymized before accessing for analysis.

All patients had some form of prior curative-intent treatment (RP, EBRT, BT, High-Intensity Focused Ultrasound (HIFU)). Patient data were collected from the electronic patient record and included age, Gleason score, TNM-classification [25], Prostate-Specific Antigen (PSA)-levels, imaging outcomes (both of the ^18^F-DCFPyL PET/CT and of subsequent imaging studies), pathology results and clinical follow-up after the PSMA PET/CT scans. BCR was defined as rising PSA-values after RP, or any PSA-level 2.0 ng/mL above the nadir following EBRT, BT or HIFU [12, 26–29].

### Imaging

^18^F-DCFPyL was synthesized via direct radiofluoration at the on-site cyclotron facilities of both hospitals, as previously described [30, 31]. Imaging was performed with a Philips Ingenuity TF (Philips Healthcare®, the Netherlands/USA) PET/CT system and a Siemens Biograph-16 TruePoint (Siemens Healthineers®, Germany). PET images were made 120 min post injection of a median dose of 307 MBq ^18^F-DCFPyL (interquartile range (IQR) 283–323 MBq). PET images were combined with a low-dose CT or a contrast-enhanced CT scan (30-110mAs, 110-130kV). All images were corrected for scatter, decay, and random coincidences; attenuation correction was performed using CT images.

### Image interpretation

Scan interpretation was performed in the participating centers by nuclear medicine physicians with experience in PCa PET reading (>300 scans). Dual-reading was performed for all scans. The final conclusion was drawn up by consensus. A scan was considered ‘positive’ if at least one lesion was suggestive for PCa recurrence (i.e., focal, tracer uptake exceeding the direct background, incompatible with physiological uptake). Loco-regional lymph node metastases were defined as lymph nodes in the true pelvis (N1), whereas lymph nodes cranial to the bifurcation of the common iliac artery were considered distant lymph node metastases (M1a).

Both visual and semi-quantitative methods for reporting the ^18^F-DCFPyL PET/CT scans were used. During the inclusion period of this study (2016–2019), several different reporting systems were adopted, including PROMISE [32] and PSMA-RADS [33]. Early on in the inclusion period, reporting was generally in line with Rauscher et al. [34].

### Verification and characteristics of ^18^F-DCFPyL detected PCa lesions

To evaluate if ^18^F-DCFPyL PET-avid lesions represented a high probability of PCa recurrence, lesions had to be verified by an additional/subsequent diagnostic study. A PET-detected lesion was considered ‘true positive’ (i.e. confirmed to be PCa) or ‘false positive’ (i.e. not confirmed to be PCa) using a composite validation method, as previously validated [14, 35, 36]. This composite validation method consisted of any of the following:

1) histopathological evaluation (lymph node dissection; CT-guided biopsy; transrectal prostate biopsy);
2) additional imaging studies (e.g. MRI, diagnostic CT scan) performed within three months from the primary ^18^F-DCFPyL PET/CT;
3) PSA-response after targeted radiotherapy (e.g. local salvage radiation therapy, metastasis directed radiation therapy). A ≥50% decrease was considered as true positive [14], in absence of additional systemic therapy).

A maximum of 5 positive lesions per organ site (e.g., lymph nodes; bone) was listed, to prevent overrepresentation of single patients with multiple metastases. Furthermore, for any additionally detected lesion above n = 5 confirmed metastases (high volume, metastatic disease), no changes in therapeutic decisions were expected.

The CT scan (low-dose or diagnostic) concomitantly performed with every PET acquisition (for anatomical correlation and attenuation correction) is known to have a limited sensitivity for detecting metastases [37, 38]. Hence, the absence of a suspicious substrate (usually based on size for lymph nodes and presence of sclerotic/lytic lesions for bones) on CT does not necessarily mean that a PET-avid lesion is falsely positive. The probability that PET-positive lesions were malignant was strengthened by the concomitant CT if one of the following findings was present: lymph nodes >6mm in size [39, 40]; and/or bone lesions with a sclerotic or lytic substrate; and/or visceral lesions suspicious for metastases (i.e., hypodense hepatic lesions incompatible with benign entities like cysts, hemangiomas, etc.). Since there is a significant overlap in the size of metastatic and non-metastatic lymph nodes the best cut-off value remains a matter of debate and different size criteria are applied in clinical practice. It has been reported that a short-axis diameter larger than 6 mm on CT-images of patients with prostate cancer, results in a specificity for the detection of a malignant nodes of 97% [39]. If the threshold is changed to a short-axis diameter larger than 5 mm for metastases (in pelvic malignancies), the specificity drops to 78% [41]. Therefore, in this study a cut-off value of larger than 6 mm was used for determination of malignant nodes on CT.

In current clinical practice, PET scans are usually assessed visually or by using semiquantitative measurements of tracer accumulation (Standardized Uptake Value; SUV). These PET-characteristics of true positive lesions were compared to false positive lesions (both validated by the composite validation method). All lesions were semi-automatically delineated using in-house developed software (Accurate-tool [42] and Syngo.via (Siemens Medical Solutions, Malvern, PA, USA). Both SUV_peak_ (maximum average SUV within a 1 cm^3^ spherical volume) and tumor to blood ratio (TBR) [43] were determined. SUV was normalized to body weight.

### Statistical analysis

Statistical analyses were performed using the Statistical Package for Social Sciences (SPSS, IBM; v25). Lesion detection rate was calculated for the following PSA strata (<0.5; 0.5-<1.0; 1.0-<2.0; ≥2.0 ng/mL) and included a 95% confidence interval (CI).

The diagnostic certainty of ^18^F-DCFPyL PET-detected lesions was calculated as the number of true positive lesions / (true positive lesions + false positive lesions).

Additionally, the diagnostic certainty was evaluated for patients with: (i) a PSA <2.0 ng/mL and ≥2.0 ng/mL (including 95%CI), (ii) in patients with CT-correlation and without CT-correlation, (iii) in patients with a SUV_peak_ <3.5 and ≥3.5 and (iv) in patients with 1 or 2 PET-positive lesions and patients with more than 2 PET-positive lesions. Differences between subgroups were tested using Fisher’s Exact test, with significance set at *P* <0.05 [44]. Differences in the SUV_peak_ and TBR between the true positive lesions and false positive lesions were determined using the Mann-Whitney U test. In addition, to assess an optimal diagnostic threshold for SUV_peak_ of ^18^F-DCFPyL PET-detected lesions, we performed a receiver operating characteristic (ROC)-analysis using the Youden index [45] to determine the cut-off point.

## Results

### Patient population

**Fig 1:**
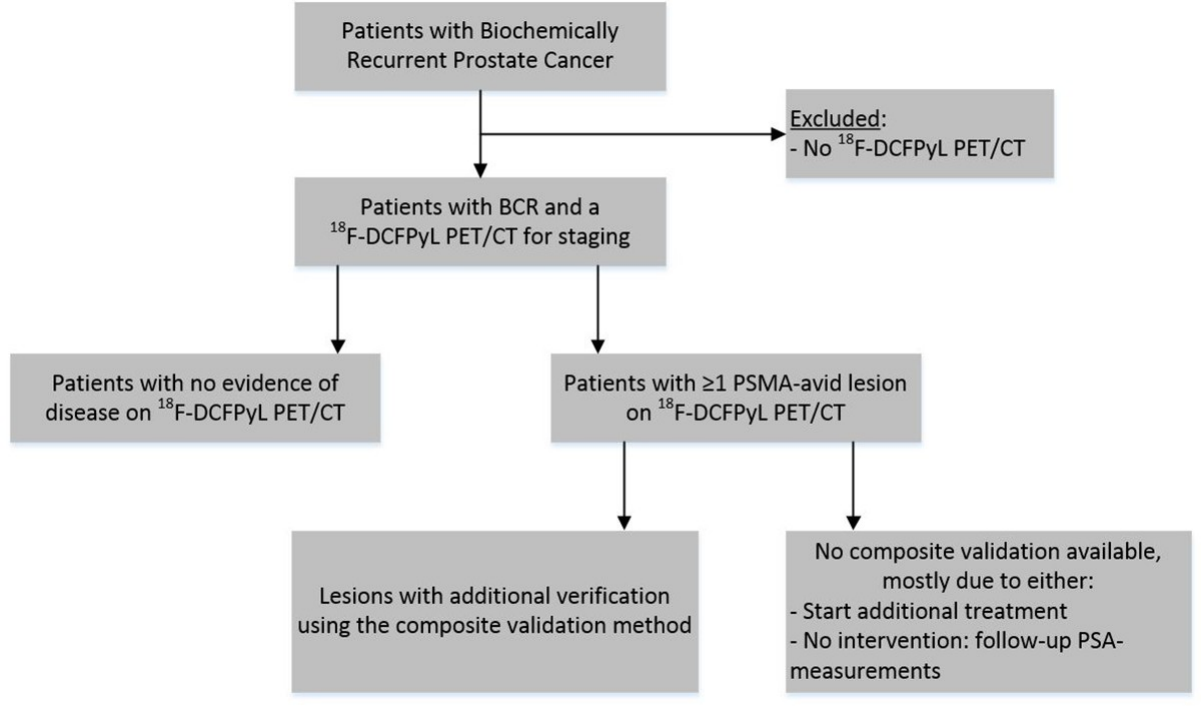
STARD flowchart of the included patients.

**Fig 2:**
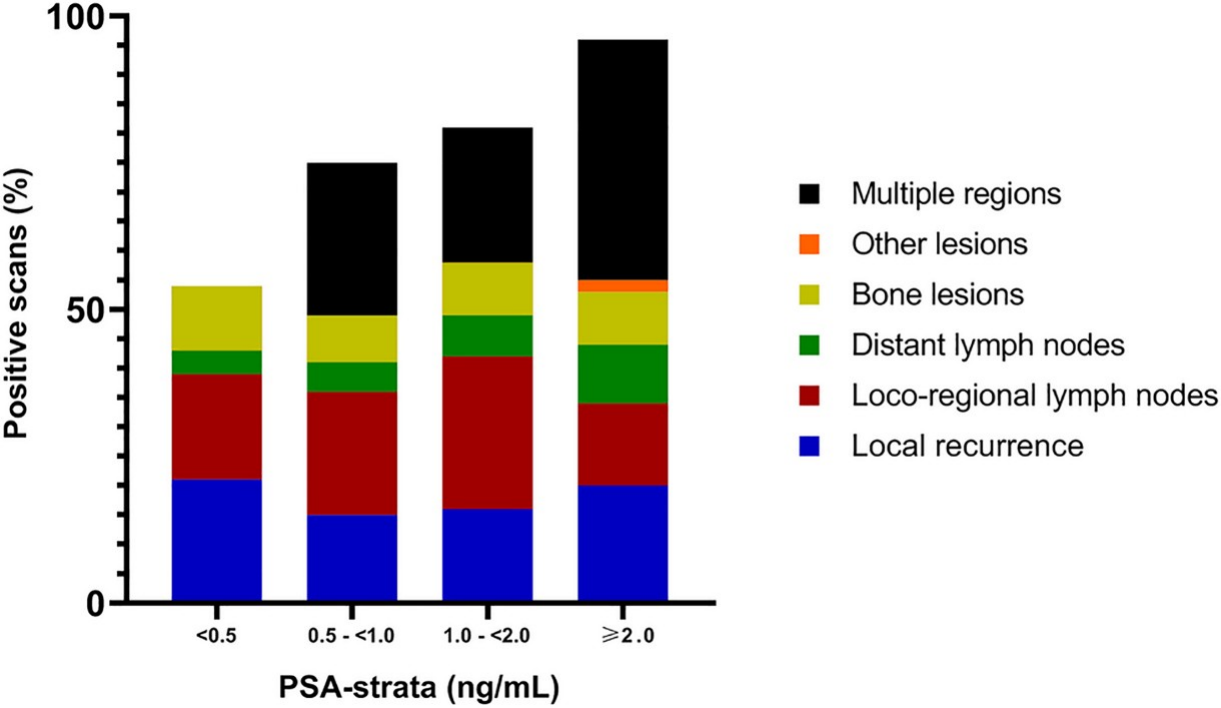
Detection rate ^18^F-DCFPyL PET/CT per PSA-strata, including localization of PET-positive lesions.

Of the 262 patients analyzed (flow chart presented in Fig 1), 127 men (48.4%) were previously treated with RP, 106 (40.5%) with EBRT, 17 (6.5%) with BT, and 12 (4.6%) with HIFU, respectively (Table 1). The median PSA-level at the time of the diagnostic PET/CT scan was 2.5 ng/mL (IQR 0.9–5.3) for all patients and 1.1 ng/mL (IQR 0.5–3.0) in patients previously treated with RP and 4.0 ng/mL (IQR 2.1–8.0) in patients treated with EBRT, BT or HIFU. In 226/262 ^18^F-DCFPyL PET/CT scans (86.3%, 95%CI 82.1–90.5%), at least one lesion suggestive of PCa recurrence was reported (‘positive scan’; Fig 2). In total 801 ^18^F-DCFPyL PET-positive lesions were reported (median 3 per positive scan, IQR 1.0–5.0).

**Table 1:** Characteristics of patients with BCR, subsequently scanned with ^18^F-DCFPyL PET/CT, stratified for their primary treatment.

	Patients with BCR after RP (n = 127)	Patients with BCR after either EBRT, BT or HIFU (n = 135)	*p*-value
Age, yrs; median (IQR)	69 (66–74)	73 (69–76)	0.001
Initial PSA-value; median (IQR)	10.7 (7.1–16.4)	17.3 (10.4–52.8)	<0.001
Tracer dose, MBq; median (IQR)	312 (290–323)	299 (275–323)	0.030
PSA at the time of PSMA PET/CT, ng/mL; median (IQR)	1.1 (0.5–3.0)	4.0 (2.1–8.0)	<0.001
**Gleason score, n (%)**			
6	9 (7.1)	31 (23.0)	0.001
7	68 (53.5)	53 (39.3)	
≥8	47 (37.0)	49 (36.3)	
Missing	3 (2.4)	2 (1.4)	
**TNM classification**			
*T-stage*, *n (%)*			
T1-T2	57 (44.9)	61 (45.2)	0.996
T3a-b	66 (52.0)	70 (51.9)	
T4a	4 (3.1)	4 (2.9)	
*N-stage after RP*, *n (%)*			
N0	61 (48.0)	-	
N+	18 (14.2)	-	
Nx	48 (37.8)	-	
*R-status after RP*, *n (%)*			
R0	63 (49.6)	-	
R1	55 (43.3)	-	
Missing	9 (7.1)	-	
**Additional treatment prior to BCR, n (%)**			
None	98 (77.2)	112 (83.0)	0.006
Hormonal treatment	8 (6.3)	16 (11.8)	
Radiation therapy	21 (16.5)	7 (5.2)	

IQR = interquartile range; MBq = megabecquerel; RP = radical prostatectomy; EBRT = external beam radiotherapy; BT = brachytherapy; HIFU = high-intensity focused ultrasound; PSA = prostate-specific antigen; PSMA = prostate-specific membrane antigen; PET = positron emission tomography; CT = computed tomography.

### Lesion validation

For 84/226 patients, composite validation of ^18^F-DCFPyL PET-detected lesions was available (37.2% of all positive scans). In the absence of composite validation, all other patients were excluded from this analysis. In the included scans, verification was available for 141 lesions (17.6% of all PET-detected lesions). From the 141 lesions, a total of 110 were considered with high probability to be PCa (78.0%, 95%CI 71.1–84.9%; Table 2), comprising 51/67 lesions post-RP (diagnostic certainty: 76.2%), and 59/74 lesions post-EBRT/BT/HIFU (diagnostic certainty: 79.7%; *p* = 0.61). When stratified for type of lesion, the diagnostic certainty of ^18^F-DCFPyL PET/CT for local recurrences was 82.8% (24/29 lesions), for lymph node metastases 70.7% (53/75 lesions) and for bone or visceral metastases 89.2% (33/37 lesions; *p* = 0.07)

**Table 2:** Diagnostic certainty of ^18^F-DCFPyL PET-detected lesions.

	Histopathology	Additional imaging	PSA-response ≥50%	Total
*True positive lesions*	24	54	32	110
*False positive lesions*	8	5	18	31
**Diagnostic certainty**	75.0%	91.5%	64.0%	78.0%

**Fig 3:**
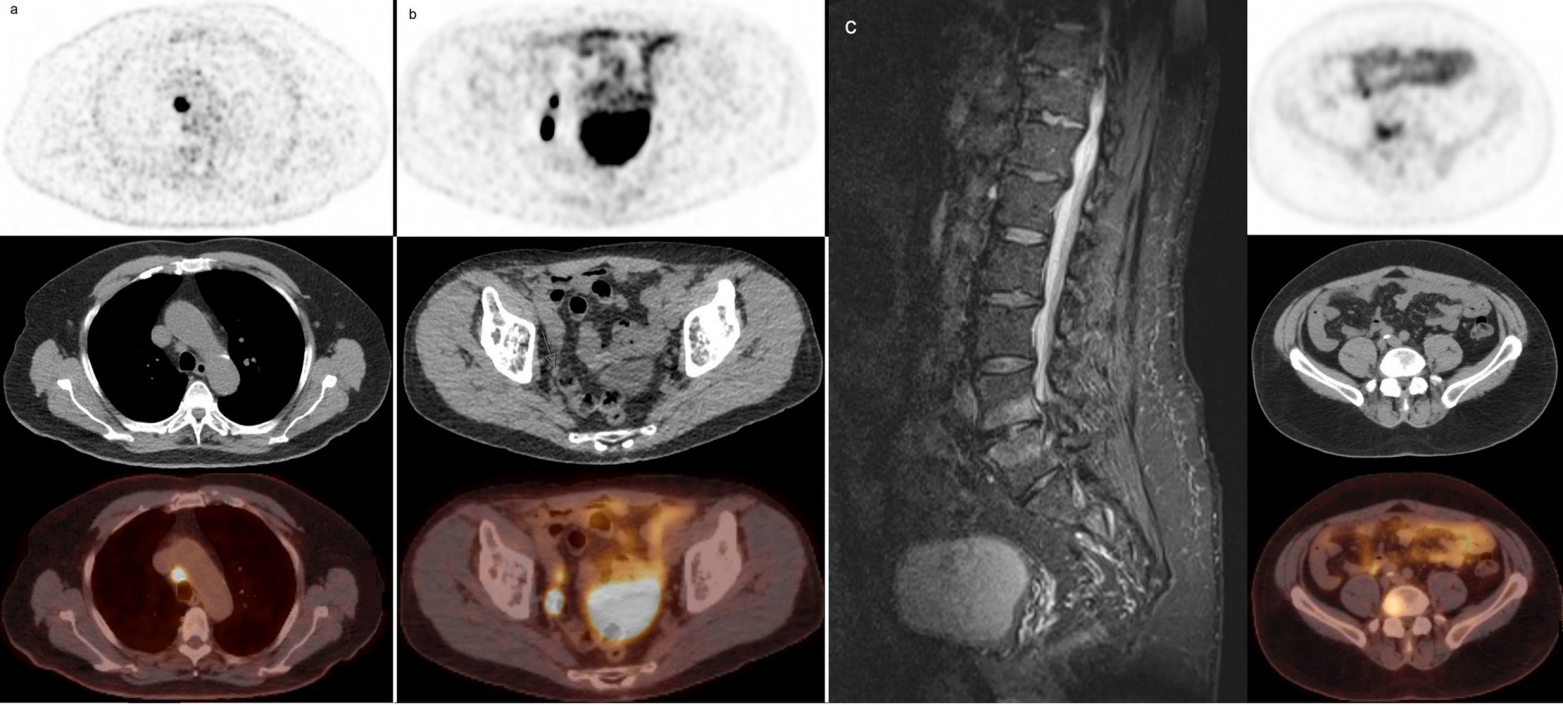
Examples of validated lesions through our composite validation method *(PET/ CT/ Fused PET/CT)*. *a*. True positive lesion: mediastinal lymph node metastasis of PCa, confirmed with endobronchial ultrasound guided biopsy. *b*. True positive lesions: two lymph nodes, proven to be metastases of PCa by extended pelvic lymph node dissection *c*. False positive lesions: high PSMA expression in sclerotic substrate lateral to the right side of the vertebral body of L4 and L5, suspicious for degenerative lesions instead of PCa metastases on additional MRI imaging.

Of the 84 patients with composite validation available, 40/226 patients (17.7%) underwent metastasis-directed radiotherapy of the PET-positive lesions. For the remaining 44/226 patients (19.5%), histopathological evaluation or follow-up imaging was present, accounting for 91 PET-positive lesions. 78/91 lesions (85.7%) were confirmed to be PCa. For the remaining 13 lesions no diagnosis of PCa could be established (Table 3). For all 8 false positive lesions upon histopathological verification, no alternative diagnoses were noted. For the 5 lesions that were not confirmed by additional imaging studies, two lesions proved to be degenerative bone changes (Fig 3).

**Table 3:** Overview of the false positive lesions, validated by either histopathology or imaging studies.

Previous curative treatment	Suspected lesion	Localization	Validation method	Result	CT-correlation	SUV_peak_
BT	Local recurrence	Prostate bed	Prostate biopsies	No prostate cancer	Not applicable	3.1
HIFU	Local recurrence	Prostate bed	Prostate biopsies	No prostate cancer	Not applicable	3.5
RP	Local recurrence	Prostate bed	Prostate biopsies	No prostate cancer	Not applicable	12.0
EBRT	LN metastasis	Para-iliac nodes	Interval PSMA PET/CT	Negative	Not applicable	0.9
EBRT	LN metastasis	Para-iliac nodes	Lymph node dissection	Normal lymphoid tissue	Negative	1.2
BT	LN metastasis	Para-iliac nodes	Lymph node dissection	Normal lymphoid tissue	Negative	1.3
RP	LN metastasis	Para-iliac nodes	MRI scan	Negative	Negative	1.5
HIFU	LN metastasis	Inguinal nodes	Lymph node dissection	Normal lymphoid tissue	Negative	1.4
RP	LN metastasis	Inguinal nodes	Lymph node dissection	Normal lymphoid tissue	Negative	1.5
BT	LN metastasis	Obturator nodes	Lymph node dissection	No prostate cancer	Negative	3.2
EBRT	Bone metastasis	Sacrum	MRI scan	Lesion not visible on MRI	Negative	1.4
RP	Bone metastasis	Vertebra L4	MRI scan	Degenerative lesion	Positive	3.9
RP	Bone metastasis	Vertebra L5	MRI scan	Degenerative lesion	Positive	3.9

CT = computed tomography; SUV = standardized uptake value; LN = lymph node; MRI = magnetic resonance imaging; PSMA = prostate-specific membrane antigen; PET = positron emission tomography.

### Diagnostic certainty

**Fig 4:**
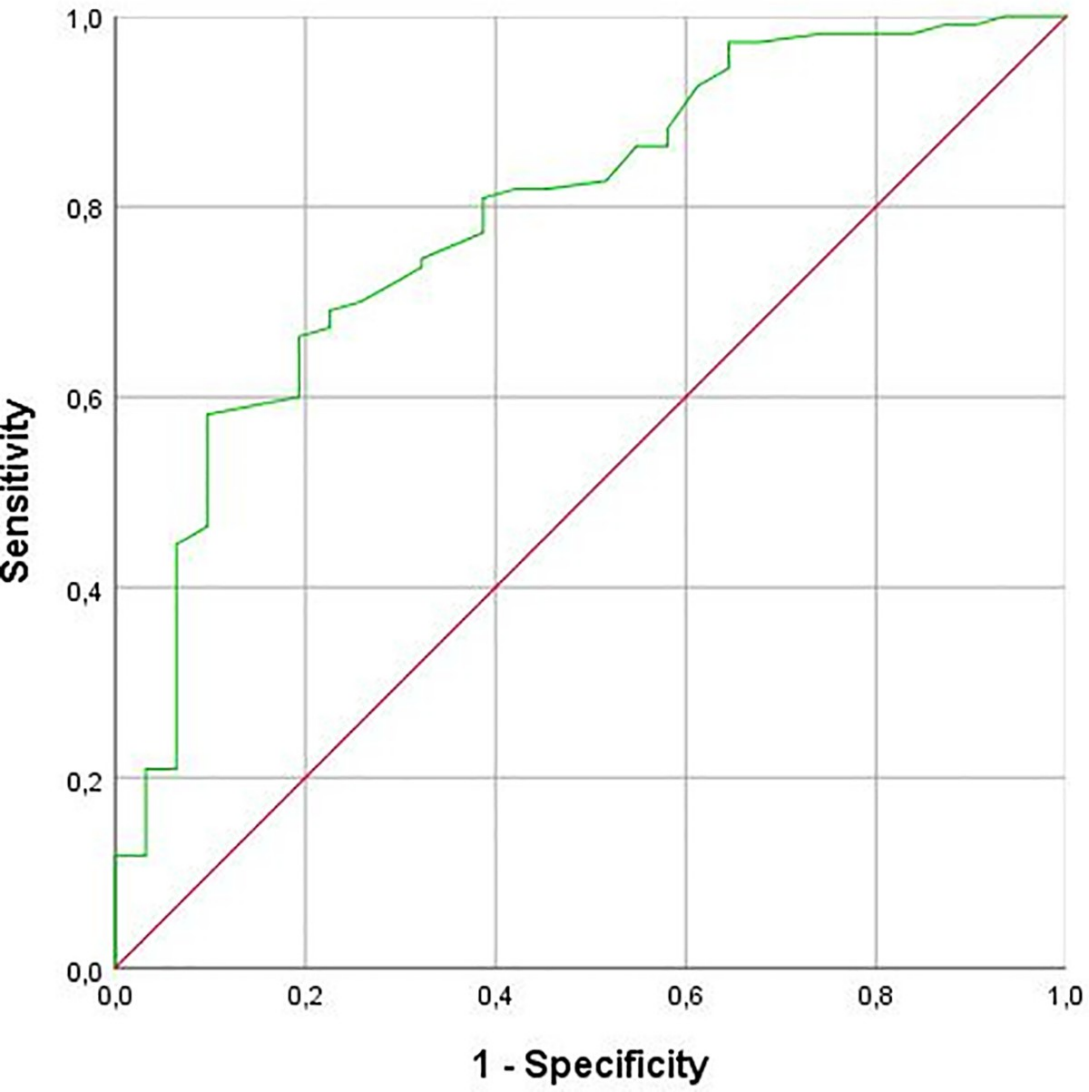
ROC-curve assessing an optimal threshold of SUV_peak_ for high diagnostic certainty of ^18^F-DCFPyL PET-detected lesions.

PET-lesions with corresponding findings on CT were considered to be malignant (PCa) in 96.5% of cases (55/57 lesions) compared to 55.6% (30/54 lesions) for lesions without any suspicious CT-findings (*p* < 0.001; Table 4). The true positive lesions showed a median SUV_peak_ of 4.8 (IQR 2.8–9.5; median TBR of 3.5 (IQR 2.1–7.2)) compared to a median SUV_peak_ of 2.4 (IQR 1.4–3.3; *p* < 0.001; median TBR of 2.3 (IQR 1.3–3.5)) for the false positive lesions. 91.4% of the lesions with a SUV_peak_ ≥3.5 were considered true malignant, versus 60.0% of the lesions with a SUV_peak_ <3.5. In lesions with a SUV_peak_ ≥4.0, the percentage of true positives was 95.5% (Table 5). Using the ROC-curve and the Youden index, the optimal cut-off appeared to be within an SUVpeak of between 3.5 and 4.0 (Fig 4).

**Table 4:** Comparison of the diagnostic certainty, stratified per clinical predictor.

	True positive	False positive	Diagnostic certainty	*p*-value
*PSA <2*.*0 ng/mL*	28	15	65.1%	0.03
*PSA ≥2*.*0 ng/mL*	82	16	83.7%	
CT-substrate -	30	24	55.6%	<0.001
CT-substrate +	55	2	96.5%	
*SUV*_*peak*_ *<3*.*5*	36	24	60.0%	<0.001
*SUV*_*peak*_ *≥3*.*5*	74	7	91.4%	
*1 or 2 PET-positive lesions*^*a*^	43	24	64.2%	0.02
*>2 PET-positive lesions*^*a*^	16	1	94.1%	

^*a*^ = patient-based analysis.

PSA = prostate-specific antigen; CT = computed tomography; PET = positron emission tomography; SUV = standardized uptake value.

**Table 5:** Comparison of the diagnostic certainty, stratified per SUV-value.

	True positive	False positive	Diagnostic certainty	*p*-value
***SUV***_***peak***_				
*<2*.*5*	20	18	52.6%	<0.001
*≥2*.*5*	90	13	87.4%	
*<3*.*0*	28	21	57.1%	<0.001
*≥3*.*0*	82	10	89.1%	
*<3*.*5*	36	24	60.0%	<0.001
*≥3*.*5*	74	7	91.4%	
*<4*.*0*	46	28	62.2%	<0.001
*≥4*.*0*	64	3	95.5%	
*<4*.*5*	52	28	65.0%	<0.001
*≥4*.*5*	58	3	95.1%	
*<5*.*0*	57	28	67.1%	<0.001
*≥5*.*0*	53	3	94.6%	

SUV = standardized uptake value.

One of the patient related factors that was associated with confirmed malignancy included a PSA ≥2.0 ng/mL (82/98 lesions confirmed, 83.7%) compared to patients with a PSA <2.0 ng/mL (28/43 lesions, 65.1%; *p =* 0.03). In addition, in patients with more than 2 PET-positive lesions, PCa could be confirmed in 94.1% (16/17 patients) compared to 64.2% (43/67 patients; *p* = 0.02) in patients with only 1 or 2 PET-positive lesions (Table 4).

For 337 of 801 PET-detected lesions, correlation on the concomitant CT-scan was present (42.1%; n = 137 enlarged lymph nodes; n = 158 bone lesions suspicious for metastases; n = 42 visceral lesions suspicious for metastases). Abnormalities compatible with malignancy were found in 250/485 lesions (51.5%) with a diagnostic CT, compared to 87/210 lesions (41.4%) with a low dose CT (*p* = 0.02). Local recurrences in the prostate bed were excluded from this analysis, as they are generally suboptimally evaluated on CT.

## Discussion

While multiple publications have reported the detection rate of PSMA PET in patients with BCR, further verification of PET-detected lesions is often lacking [14, 19, 22–24, 46, 47]. A high true positive rate is becoming more important in clinical practice, both for the detection of metastases (e.g. due to the emergence of strategies like local, metastasis-directed therapy) as well as the primary tumors [48]. Therefore, the purpose of this study was to evaluate ^18^F-DCFPyL PET-detected lesions in patients with BCR and identify clinical/imaging characteristics that increase diagnostic certainty regarding the malignant nature of PSMA-avid lesions.

In patients with BCR and low PSA-values, lesions are often smaller than 1 cm, and their location may be unfavorable, making biopsy procedures more difficult. Another reason to forego verification of lesions in patients with BCR may be the rather promising specificity of PSMA PET/CT (97%) demonstrated in patients with primary PCa who undergo lymph node dissection [49]. However, some caution is necessary in extrapolating from the primary to recurrence/progression settings, since the diagnostic accuracy of PSMA PET can differ.

A recent meta-analysis evaluating ^68^Ga-PSMA PET showed detection of metastases in 80–90% in patients with BCR, whereas the reported sensitivity in primary staging appears significantly lower (40–50%) [47]. This apparent difference means that thorough analysis of PET-detected lesions in patients with BCR is warranted, as a higher sensitivity might increase the number of false positives. Identifying a lesions as false positive could have a significant impact on management decisions. Therefore, we retrospectively verified the nature of ^18^F-DCFPyL PET-detected lesions in 262 patients with BCR for which any form of validation was present.

A significant proportion of patients who underwent a RP, did not undergo a concomitant (extended) pelvic lymph node dissection and yet more than 50% of patients had evidence of extraprostatic extension (≥pT3) on histopathological evaluation. This apparent paradox can be explained by the fact that the decision to perform a radical-intent prostatectomy was usually made based on the clinical T-stage, which can underestimate the true (pathological) T-stage [50]. Therefore, a significant number of patients with ≥pT3 disease were in retrospect understaged pre-operatively, and consequently, did not undergo an extended pelvic lymph node dissection.

Lesions suggestive of PCa were found at all PSA-levels, with, overall, 86.3% (95% CI 82.1–90.5%) of ^18^F-DCFPyL PET/CT scans being reported as positive. This finding appears similar to other recent studies [13, 22, 47]. Additional diagnostic procedures were available to verify the nature of the PSMA-avid lesions in a minority of cases (37.1% of all patients). For all lesions for which composite validation was available, 78.0% were confirmed to be PCa.

To help improve ^18^F-DCFPyL PET/CT reporting, we aimed to identify the scan parameters associated with a high probability of PCa (true positives). Of all PET-positive lesions, 50% had corresponding CT findings (i.e. lymph nodes >6mm in size; and/or bone lesions with sclerosis or lytic substrate; and/or visceral lesions suspected for metastases). The majority of these abnormalities on CT were found using a contrast-enhanced diagnostic CT. In the presence of a characteristic abnormal CT substrate, we found that 97% of the PET-detected lesions were true positives. This would support the need to perform a diagnostic concomitant CT instead of a low-dose CT-scan.

Tracer uptake (both SUV_peak_ and TBR) of the true positive lesions was significantly higher than false positive lesions. We assessed an optimal threshold of SUV_peak_ for a high diagnostic certainty of ^18^F-DCFPyL PET-detected lesions, using the receiver operating characteristic (ROC)-curve (Fig 4) and the Youden index. The highest Youden index appeared to be within an SUV_peak_ of between 3.5 and 4.0. Combining this with the data in Table 5, we found an optimal classification threshold at SUV 3.5, with a diagnostic certainty for these lesions >90%. Furthermore, in patients with a PSA-value ≥2.0 ng/mL, lesions were significantly more often confirmed to be PCa. In addition, we demonstrated that patients with more than 2 PET-positive lesions were more likely to have proven PCa.

The results should be interpreted as reflecting current clinical practice at our institutes. In daily clinical care, validation (biopsy or additional imaging) is most likely to be deemed necessary for equivocal lesions only. It is possible (e.g. due to limitations in surrogate markers of positivity such as lymph node size) that the overall diagnostic performance of ^18^F-DCFPyL is higher. Since in clinical practice, nuclear medicine physicians may sometimes encounter difficulty in interpreting suspicious PSMA uptake, the presented findings provide several predictors of true positive PCa detection in patients with BCR. To facilitate uniform image reporting, several standardized classification systems have been proposed, such as the PSMA-RADS and the PROMISE criteria [32, 33]. However, clinical diagnostic reporting tools to characterize PSMA PET-equivocal lesions are still lacking.

This study is inherently limited by its retrospective nature. Most importantly, this means no strict protocol for the verification of PSMA-avid lesions was followed. Our aim was not to provide a general estimate of the validity of ^18^F-DCFPyL-findings since this would require systematic evaluation of all lesions. Hence, we refrained from calculating a general *positive predictive value* for ^18^F-DCFPyL PET/CT.

Regarding histopathologic evaluation, a few methodological limitations need to be addressed. Firstly, the chance of labelling lesions as false positive rises if the biopsy result itself is false negative. This may have been the case in some patients, resulting in an inflated number of false positive lesions after histopathological verification. On the other hand, not all lesions were suitable for biopsy. Small PSMA-avid lymph nodes were not biopsied and therefore not included in the histopathological verification arm of this study, possibly resulting in a slight overestimation of the diagnostic certainty.

Currently, there is limited literature on the specificity of ^18^F-DCFPyL PET in patients with BCR. A recent abstract with preliminary results of a prospective trial in 93 patients with BCR and known metastatic disease is notable [51]. In contrast to the current analysis, these patients had histopathologic evaluation of all detected lesions, and a mean PPV of 84.5% was reported. Given the difference in methods, these results appear broadly in line with the present findings.

## Conclusion

In this study, clinical verification of ^18^F-DCFPyL PET-positive lesions in patients with BCR was performed. In addition to a high detection rate, ^18^F-DCFPyL PET/CT appears to be both a sensitive and reliable instrument to guide further treatment in patients with BCR. Diagnostic certainty of detected lesions increases in the presence of characteristic abnormalities on CT, when SUV_peak_ is ≥3.5, when PSA-levels exceed 2.0 ng/mL or in patients with more than two PET-positive lesions. These parameters should be validated in future prospective trials.

## Supporting information

S1 Data (XLSX)
